# PERCEIVED WORKPLACE AGEISM AND RACIAL/ETHNIC DISPARITIES IN COGNITIVE FUNCTIONING AMONG OLDER WORKERS

**DOI:** 10.1093/geroni/igad104.0875

**Published:** 2023-12-21

**Authors:** Duygu Basaran Sahin, Frank Heiland, Na Yin, Ryan Smith

**Affiliations:** RAND Corporation, Brooklyn, New York, United States; Baruch College, New York, New York, United States; Baruch College, CUNY, New York City, New York, United States; Baruch College, New York, New York, United States

## Abstract

Research suggests that discrimination contributes to racial/ethnic disparities in health, but little is known about the role of perceived workplace ageism in this phenomenon. Workplace discrimination threatens a person’s livelihood, self-esteem, and identity. Older workers from racial/ethnic minority groups may experience both race- and age-based discrimination. Using data from the Health and Retirement Study, we investigate whether workplace ageism is related to older workers’ cognition and whether the link differs by race/ethnicity. We find that workers who perceive ageism in their workplaces tend to do worse on standard cognition tests than those who do not. For example, the immediate and delayed word recall performance is 13-14% of one SD lower among individuals who report that co-workers pressure older workers to retire before age 65. The relationships are stronger (more negative) for Hispanic workers, but there is no evidence that they differ between Non-Hispanic Black and Non-Hispanic White workers.

